# The System 3 Method: integrating intuition, reason and creative metacognition for deep learning

**DOI:** 10.3389/fpsyg.2026.1891793

**Published:** 2026-07-24

**Authors:** Juan Carlos Chávez-Autor

**Affiliations:** 1 College of Psychology, Keiser University, Fort Lauderdale, FL, United States; 2 Facultad de Comunicación, Universidad Panamericana, Mexico City, Mexico; 3 Bio-Intelligence & Creativity Institute, Playa del Carmen, Mexico

**Keywords:** creative metacognition, deep learning, educational psychology, generative learning, human–AI learning, intuition–reason integration, metacognitive control, System 3 Method

## Abstract

Educational psychology increasingly needs frameworks that connect deep learning, generative learning, learner agency, creative metacognition, and metacognitive control into a coherent account of how students learn. This Conceptual Analysis introduces the System 3 Method, a pedagogical framework in which learners are guided through iterative cycles that integrate intuitive salience and rational articulation under implicit and explicit metacognitive control. The article clarifies that System 3 is not an anatomical module and not a synonym for metacognition. It is defined as a functional coordination construct: the regulated coupling through which learners shift among intuitive orientation, generative construction, rational verification, integration, and transfer. The method translates this construct into GRIM^T^: Generation, Reflection, Integration/Iteration, Metacontrol, and Time. It further proposes NUD—novelty, usefulness, and diversity—as a complementary profile for evaluating creative learning trajectories alongside academic mastery, with novelty understood primarily as learner-relative mini-c creativity. The revised analysis situates the method more explicitly in relation to self-regulated learning, ICAP, generative learning, curiosity-driven conceptual change, knowledge integration, and learner-centered pedagogies. It also provides a worked instructional example, clarifies developmental and domain-specific boundary conditions, and distinguishes content-relevant meaningfulness from merely interesting or seductive details. Technology and artificial intelligence are discussed as possible generative scaffolds, adaptive critics, metacognitive mirrors, and process memories only when a human gate preserves authorship, verification, persistence, and transfer. The central contribution is a falsifiable framework for a pedagogy of creative metacognition: education should train learners not only to know more, but to participate more intelligently in constructing, evaluating, integrating, and transforming what they know.

## 1 Introduction: why deep learning requires a System 3 account

Contemporary education faces a paradox. Learners have unprecedented access to information, explanations, simulations, and intelligent tools, yet access alone does not guarantee understanding. A student may recognize terms without integrating them, complete tasks without transferring them, or receive personalized content without developing the capacity to regulate learning independently. In this context, the central challenge for educational psychology is not merely how to deliver more information, but how to cultivate learners who can transform information into meaningful, durable, transferable, and self-regulated understanding. Deep learning, in this sense, is the learner's participation in the construction, evaluation, integration, and transfer of meaning.

This challenge is not new, but it is becoming more urgent. Constructivist, sociocultural, self-regulated learning, and metacognitive traditions have long emphasized that learners actively organize knowledge rather than passively absorb it (Vygotsky, 1978; Flavell, 1979; Zimmerman, 2002). Research on generative learning has shown that students learn more deeply when they select, organize, explain, draw, teach, or otherwise transform material rather than merely receive it (Fiorella and Mayer, 2016; Mayer, 2020). Creativity research has likewise demonstrated that productive thought depends on both generative expansion and evaluative constraint (Runco and Jaeger, 2012; Sternberg and Lubart, 1995). However, these literatures often address different levels of the learning problem. Self-regulation describes cycles of forethought, performance, monitoring, and reflection; ICAP differentiates modes of engagement; generative learning identifies productive strategies; and knowledge-integration traditions emphasize the construction of connected representations. What remains under-specified is how intuitive salience, rational articulation, creativity, feedback, and metacognitive control are coordinated within a single instructional episode.

The System 3 Method is proposed as a conceptual bridge across these domains. It builds on the claim that human creativity and deep learning cannot be adequately understood as the output of either fast intuition or slow reasoning alone. In dual-process terms, System 1 is commonly associated with rapid, associative, affect-laden, and low-effort processing, whereas System 2 is associated with slower, deliberative, rule-based, and effortful reasoning (Kahneman, 2011; Evans and Stanovich, 2013). Both are essential for learning. Intuition orients attention, marks relevance, supports pattern recognition, and provides affective momentum. Reason articulates, tests, justifies, revises, and constrains. Educationally powerful learning requires more than alternation between these modes. It requires a higher-order integrative process that monitors, coordinates, and iteratively binds intuitive and rational signals into meaningful understanding.

We refer to this higher-order integrative process as System 3. In the present article, System 3 is not defined as a third anatomical module or a replacement for dual-process theory. It is a functional coordination construct: an emergent, metacognitively governed mode of cognition in which intuitive and rational processes are iteratively integrated under implicit and explicit metacognitive control. In creativity, this process has been described as a generate-evaluate-gate cycle: intuitive mechanisms generate candidate associations or possibilities; analytic mechanisms evaluate them against constraints; and a metacognitive gate regulates whether to explore further, revise, integrate, or commit (Chávez-Autor, 2025). Translated into education, this architecture suggests that learning becomes deeper when students do not merely receive correct representations, but repeatedly construct, test, integrate, and reflect on representations in ways that make them personally meaningful and conceptually accountable.

The System 3 Method can therefore be defined as a pedagogical framework for deep and transcendent learning that guides learners through iterative cycles in which intuition and reason are integrated under implicit and explicit metacognitive control, transforming educational content, affective relevance, feedback, and reflection into meaningful, transferable, and creatively regulated understanding over time. Here, transcendent learning does not imply a metaphysical claim. It refers to learning that exceeds immediate task performance by becoming transferable across contexts, integrated with the learner's self-regulatory capacities, and formative for future thought. The guiding proposition is simple: learners remember more deeply what they help make meaningful.

This article advances a Conceptual Analysis of the System 3 Method for educational psychology. It does not report new empirical data. Instead, it offers a theoretical framework, a pedagogical architecture, a worked instructional contrast, observable indicators, and a research agenda. The argument proceeds in five movements: first, it situates System 3 learning within dual-process cognition and metacognition; second, it defines the method and its distinct contribution relative to existing frameworks; third, it develops the learning cycle, developmental calibration, and assessment through GRIM^T^ and NUD; fourth, it clarifies the role of AI and the relation to learner-centered traditions; and fifth, it identifies boundary conditions, testable propositions, and implications for a pedagogy of creative metacognition.

## 2 Conceptual foundation: from dual-process cognition to System 3 learning

Dual-process theories provide a useful starting point for understanding learning, but not a complete architecture for deep learning. In their most influential formulation, fast, autonomous, associative processes are contrasted with slower, deliberative, working-memory-dependent processes that support hypothetical thinking, rule use, and cognitive control (Evans and Stanovich, 2013; Kahneman, 2011). In educational contexts, this distinction clarifies why learners often respond first through familiarity, salience, affect, fluency, or pattern recognition, and only later through explanation, verification, and justification. However, the same distinction can become misleading if interpreted as a hierarchy in which intuition is merely an error-prone source of bias and reason is merely a corrective mechanism. Learning begins before formal explanation. Selective attention, curiosity, confidence, confusion, bodily orientation, and affective relevance shape what becomes available for later reasoning through partly distinct mechanisms: interest supports engagement (Hidi and Renninger, 2006), curiosity can enhance information seeking and memory (Kang et al., 2009), task-relevant confusion may promote deeper processing when appropriately resolved (D'Mello et al., 2014), and confidence/error signals can influence subsequent cognitive control (Bang et al., 2018; Boldt and Yeung, 2015).

For the purposes of the System 3 Method, intuition refers to the rapid, affect-laden, and associative orientation through which learners first detect relevance, possibility, uncertainty, or meaning. This is not treated as irrationality, but as an initial regulatory signal. Feelings and affective states are mental experiences of bodily states that participate in life regulation and can guide attention, valuation, and choice under uncertainty (Damasio and Carvalho, 2013). Reason, by contrast, refers to the slower set of processes through which learners articulate causes, test claims, compare alternatives, inhibit misleading responses, and align understanding with evidence or task constraints. Executive functions such as updating, shifting, and inhibition are strongly implicated in intelligence and creativity, suggesting that creative learning depends not only on idea generation but also on controlled refinement (Benedek et al., 2014).

Creative cognition research reinforces this dual requirement. Creative production is associated with both associative generation and executive evaluation, and high-quality creative thought appears to depend on flexible cooperation between brain networks involved in internally generated thought and those involved in cognitive control (Beaty et al., 2015, 2016). Dual-process analyses of creativity similarly suggest that generative and evaluative modes are shifting, recurrent forms of thought that interact during creative problem solving (Sowden et al., 2015). The educational analog is not that every lesson must resemble artistic invention, but that deep learning requires a regulated alternation between opening possible meanings and constraining them through explanation, evidence, and feedback.

System 3 is proposed as the integrative mode that coordinates these processes. It is broader than metacognition, but it cannot exist without metacognition. Metacognition refers to knowledge and regulation of one's own cognitive processes (Flavell, 1979), including monitoring and control at the meta-level (Nelson and Narens, 1990). System 3 refers to the functional configuration in which that monitoring and control regulate the coupling between intuitive salience and rational articulation. Thus, metacognition is the control capacity; System 3 is the coordinated learning mode produced when metacognition governs the interplay of intuition, generation, evaluation, integration, and transfer. This distinction makes the label more than a synonym: System 3 is not simply “thinking about thinking”; it is the regulated transformation of intuitive and rational representations into transferable understanding.

This construct can be studied empirically without assuming a dedicated neural module. Researchers can examine behavioral indices such as transitions between exploratory and evaluative utterances, explanation revisions after feedback, confidence calibration, delayed transfer, and changes in representational diversity. Neural work on decision confidence and metacognitive ability suggests plausible control inputs for such regulation (Fleming and Dolan, 2012; Bang et al., 2018), while network studies of creativity indicate that integration across associative and executive systems can support high-quality idea production (Beaty et al., 2015, 2016). The educational implication is that a learning environment should not merely ask students to be intuitive, rational, creative, or reflective. It should train adaptive transitions among these modes: when to open exploration, when to narrow verification, when to revise, and when to transfer. Successful transition training would be observed not in more frequent switching *per se*, but in better-calibrated switching: faster recognition of impasse, more appropriate requests for evidence, improved confidence calibration, and stronger delayed transfer.

Accordingly, System 3 learning can be stated as follows: deep learning emerges when intuitive salience, rational articulation, feedback, and metacognitive regulation are repeatedly integrated into increasingly transferable understanding. This preserves the strengths of dual-process theory while addressing its educational limitation: the deepest learning does not occur in System 1 or System 2 alone, but in their regulated and repeated integration.

## 3 Defining the System 3 Method and its added explanatory value

The System 3 Method is not a curriculum, a single instructional technique, or a technological product. It is a pedagogical framework for organizing learning experiences around the iterative integration of intuition, reason, feedback, and metacognitive regulation. It can be formally defined as follows: the System 3 Method is a pedagogical framework for deep and transcendent learning that guides learners through iterative cycles in which intuition and reason are integrated under implicit and explicit metacognitive control, transforming educational content, affective relevance, feedback, and reflection into meaningful, transferable, and creatively regulated understanding over time. This emphasis is consistent with the distinction between rote and meaningful learning (Mayer, 2002), while specifying the coordination processes through which meaningful understanding may be constructed.

The explanatory value of the method depends on what it adds to existing frameworks. Zimmerman's self-regulated learning model explains how learners set goals, monitor performance, and reflect on outcomes (Zimmerman, 2002). ICAP differentiates passive, active, constructive, and interactive modes of engagement (Chi, 2009). Generative learning theory identifies strategies such as self-explaining, drawing, mapping, and teaching (Fiorella and Mayer, 2016). The SEEK model explains curiosity as a catalyst for conceptual change through schema expansion and error-driven knowledge building (Xu et al., 2026a). Knowledge integration and practicing connections frameworks emphasize connecting prior and new ideas into coherent structures (Linn et al., 2004; Fries et al., 2021). The System 3 Method does not replace these frameworks. Its proposed contribution is to specify the moment-to-moment coordination problem that cuts across them: how affectively marked relevance, learner generation, rational verification, integration, and metacognitive gating should be organized into a recurrent instructional cycle. Table 1 summarizes these conceptual contrasts.

**Table 1:** Conceptual contrasts between related frameworks and the System 3 Method.

Framework	Primary contribution	System 3 addition
Self-regulated learning	Goal setting, monitoring, strategy use, reflection across learning episodes.	Specifies how metacognitive control regulates the transition between intuitive salience, generation, verification, and integration within an episode.
ICAP	Differentiates passive, active, constructive, and interactive engagement.	Predicts that constructive activity is strongest when paired with verification, integration, and calibration, not merely when overt activity is present.
Generative learning and knowledge integration	Provides strategies and emphasizes connected representations.	Adds a gating architecture for when to open generation, constrain it, and bind intuitive and rational codes over time.
SEEK/curiosity-based models	Explains curiosity-driven schema expansion and inquiry.	Treats curiosity as one relevance signal inside a broader cycle that also requires rational articulation, feedback, and transfer.

This distinction matters because two learning activities can be equally active but different in System 3 quality. A student may complete many constructive actions without monitoring why an idea attracted attention, how it was verified, or how feedback changed the representation. Conversely, a reflective student may monitor performance without generating or integrating alternative representations. System 3 predicts that the strongest delayed transfer should occur when generative construction, verification, integration, and metacognitive calibration are jointly present. This is a sharper prediction than the claim that active learning is beneficial in general.

The method also clarifies the relation between creativity and deep learning. Creative cognition is not necessary for every form of learning if creativity is defined at the societal level. Students can learn facts, procedures, or vocabulary without producing culturally novel ideas. However, in educational settings creativity is often learner-relative. The Four-C model distinguishes mini-c creativity, or personally meaningful interpretation and transformation during learning, from little-c, Pro-c, and Big-C creativity (Kaufman and Beghetto, 2009). The System 3 Method uses creativity primarily in this mini-c sense. A learner is creative when he constructs a personally new connection, explanation, representation, analogy, or question that advances understanding. Thus, the method does not claim that all deep learning must be socially original; it claims that deep learning often benefits from mini-c transformation guided by verification and integration.

Generation is therefore also defined broadly. It includes overt production, such as drawing a model or posing a hypothesis, but also internal constructive acts such as predicting, selecting a relevant feature, reorganizing an example, or self-explaining. Deep learning can occur with limited overt generation, especially when learners integrate new ideas into prior knowledge. The System 3 claim is more constrained: when learning goals require transfer, explanation, or conceptual change, some generative or reconstructive act is likely to make integration and metacognitive monitoring more observable and trainable.

Meaningfulness must also be defined carefully. The guiding proposition that learners remember more deeply what they help make meaningful does not imply that personally interesting details automatically improve learning. Research on seductive details shows that interesting but task-irrelevant material can impair learning by activating inappropriate schemas or imposing extraneous processing (Harp and Mayer, 1998). In the System 3 Method, meaningfulness is content-relevant integration: a learner-selected or learner-generated element becomes meaningful only when it is connected to the to-be-learned representation, subjected to verification, and used for later transfer. Personal relevance is an entry point, not a substitute for conceptual accountability.

The method is agency-supportive but not choice-maximalist. Learner agency matters because autonomy, competence, and relatedness are associated with intrinsic motivation and self-regulated development (Deci and Ryan, 2000). Yet excessive or poorly structured choice can be demotivating or burdensome (Iyengar and Lepper, 2000; Patall et al., 2008). Bounded creative choice is therefore one mechanism within System 3 learning, not its foundation. A choice becomes pedagogically valuable only when followed by elaboration, verification, integration, and reflection. The teacher is reframed as an architect of System 3 conditions: a designer of learning environments where intuitive relevance, rational explanation, feedback, and metacognitive reflection repeatedly meet.

## 4 GRIM^T^ as a pedagogical architecture

If the System 3 Method defines deep learning as iterative integration under metacognitive control, GRIM^T^ specifies the process architecture through which that integration can be designed, observed, and trained. GRIM^T^ is not a literal psychometric equation or a claim that learning can be reduced to one number. It is a pedagogical grammar: Generation, Reflection, Integration/Iteration, Metacontrol, and Time.

Generation (G) refers to the learner's production or reconstruction of possibilities. In educational settings, generation includes asking questions, predicting outcomes, producing explanations, drawing models, imagining alternatives, selecting examples, forming hypotheses, or connecting new content to prior knowledge. Generative learning research indicates that learners benefit when they mentally reorganize, explain, draw, teach, test, or otherwise generate meaning from material (Fiorella and Mayer, 2016), and the generation effect shows that learner-produced information is often remembered better than information merely presented (Slamecka and Graf, 1978). Generation opens the possibility space, but without constraint it can become dispersion.

Reflection (R) refers to focused evaluation, justification, and constraint. It is the process by which learners compare generated possibilities against evidence, rules, goals, coherence, and feedback. Reflection includes checking an answer, explaining why a claim follows, identifying an error, distinguishing familiarity from understanding, and revising a first impression. Educationally, R prevents generative activity from becoming mere fluency. It connects System 3 to active-constructive learning while emphasizing that deeper engagement requires learners to infer, explain, and repair understanding (Chi, 2009).

Integration/Iteration (I) refers to the binding and reworking of intuitive and rational codes into coherent representations. Integration occurs when affective relevance, image, narrative, concept, rule, evidence, prior knowledge, and language become connected in a single learning structure. Iteration occurs when that structure is revised through repeated cycles of generation and reflection. This component aligns with knowledge-integration and practicing-connections frameworks, which emphasize that deep understanding in complex domains depends on connecting ideas rather than storing isolated facts (Linn et al., 2004; Fries et al., 2021). In System 3, I is the hinge that turns episodes into understanding: what begins as curiosity or surprise becomes an articulated explanation, and feedback reorganizes the representation for future use.

Metacontrol (M) refers to regulation of the learning process itself. It includes implicit and explicit monitoring of confidence, confusion, effort, conflict, progress, and strategy. Successful learning depends on learners' capacity to monitor and control cognition, although these abilities vary by age, domain, task, and instructional support (Veenman et al., 2006). The System 3 Method extends this logic by placing metacontrol at the center of creative learning: students are trained to recognize whether they are exploring possibilities, verifying a claim, integrating feedback, or repeating a habit. Metacontrol also balances persistence and flexibility, two pathways that creativity and problem solving require in dynamic coordination (Hommel, 2015; Nijstad et al., 2010).

Time (T) represents structured recurrence. It is not mere elapsed time or lesson duration. It refers to the repeated enactment of meaningful GRIM cycles across episodes, tasks, and developmental stages. Deep learning is consolidated through retrieval, spacing, feedback, and iterative reconstruction. Retrieval practice improves long-term retention (Roediger and Karpicke, 2006), and distributed practice often supports retention more effectively than massed exposure (Cepeda et al., 2006). The System 3 claim is not that practicing the cycle alone creates domain-general expertise. Rather, GRIM^T^ is a domain-general control grammar whose effectiveness depends on domain-specific knowledge, criteria, representations, and feedback. A System 3 science lesson and a System 3 art lesson share the same process grammar, but differ in standards of evidence, products, tools, and what counts as a useful transformation.

The components are interdependent. High G with low R may be imaginative but inaccurate; high R with low G may be precise but rigid; G and R without I may produce and evaluate ideas without durable understanding; and G, R, and I without M may leave the learner dependent on external guidance. A learning experience is fully System 3 only when it invites students to generate or reconstruct meaning, requires reflection and verification, supports integration, makes metacognition visible, and recurs over time in ways that build transfer.

## 5 The System 3 learning cycle and a worked instructional contrast

**Figure 1:**
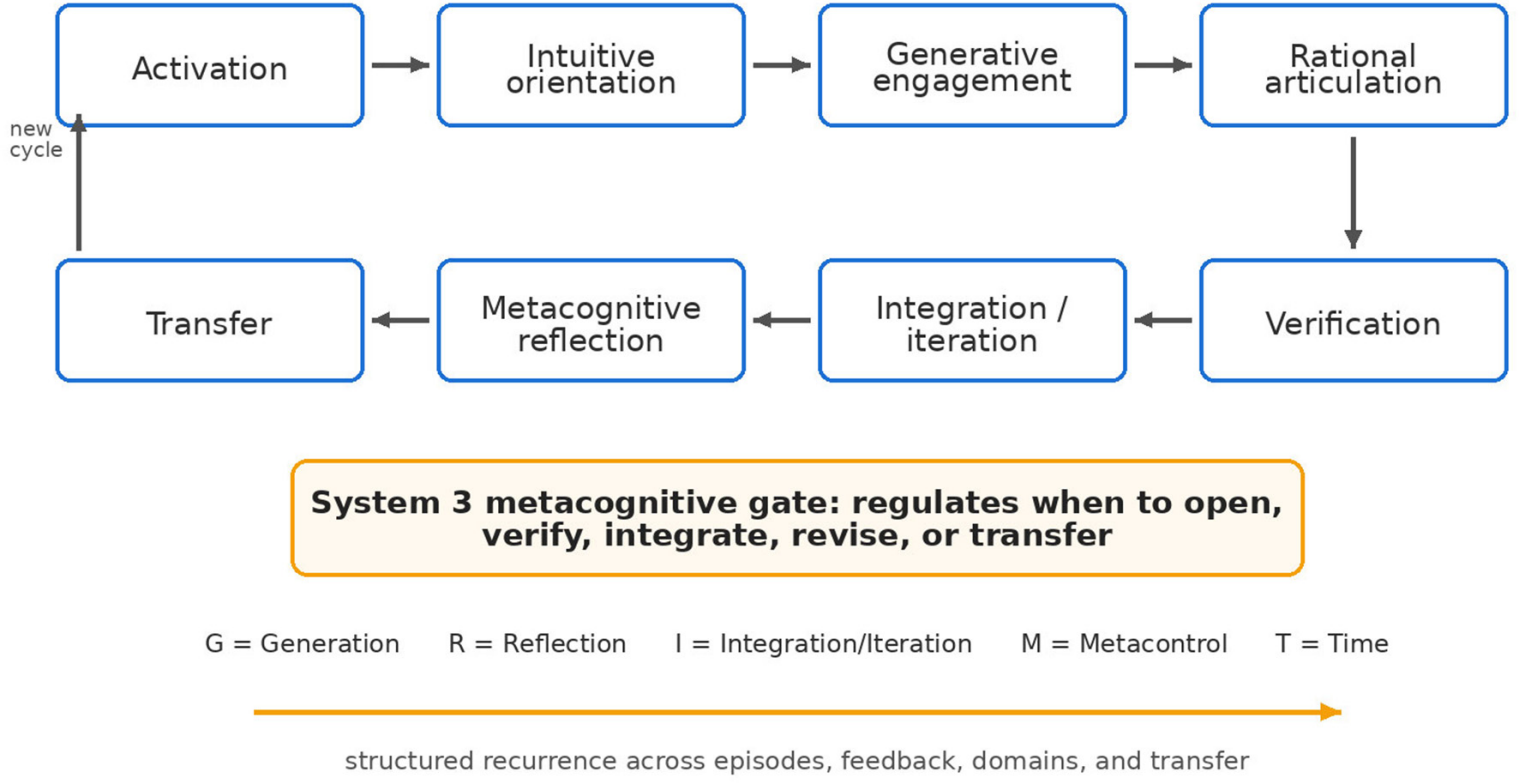
System 3 learning cycle and GRIM^T^ pedagogical architecture.

If GRIM^T^ specifies the process architecture of the System 3 Method, the System 3 Learning Cycle specifies its pedagogical sequence: Activation -> Intuitive orientation -> Generative engagement -> Rational articulation -> Verification -> Integration/iteration -> Metacognitive reflection -> Transfer. These phases are not rigid steps. Learners may move back and forth among them. Their value lies in making visible the transitions that deep learning requires: from salience to meaning, from possibility to explanation, from feedback to revision, and from task performance to transferable understanding. Figure 1 illustrates this learning cycle and its relation to the GRIM^T^ pedagogical architecture.

Activation creates a meaningful entry point into content through a question, image, object, problem, narrative, anomaly, or contrast case. The goal is not merely to capture attention, but to prepare the learner for future understanding by differentiating the knowledge space (Schwartz and Bransford, 1998). Intuitive orientation then refers to the learner's initial affective and perceptual alignment with the task. Curiosity, surprise, confusion, attraction, resistance, or a sense that “this matters” are treated as signals to be pedagogically used rather than ignored. Such signals can initiate learning, but they are not yet understanding.

Generative engagement invites learners to produce or select possible meanings. They may ask a question, predict an outcome, draw a model, choose a relevant idea, propose an explanation, form a hypothesis, or connect content to personal experience. Rational articulation then transforms possibility into explicit form: an intuition becomes a reasoned explanation, a selected idea becomes a claim, and an image becomes a model. Articulation differs from integration because it externalizes or clarifies a candidate meaning; integration binds that meaning into a broader knowledge structure. Verification introduces feedback, evidence, and constraint. The learner's explanation is checked against task goals, disciplinary standards, peer dialogue, teacher guidance, or adaptive system feedback. Feedback is most powerful when it clarifies where learners are, where they are going, and what action can reduce the gap (Hattie and Timperley, 2007).

Integration/iteration is the phase in which the learner revises and binds the experience into a more coherent representation. Metacognitive reflection makes the process visible. Importantly, metacognition is not present only at the end. Implicit metacognitive signals can operate throughout the cycle as confidence, conflict, effort, or felt uncertainty; explicit metacognitive reflection is a deliberately instructional phase in which students name and evaluate the process. Transfer completes the cycle by moving understanding beyond the original task. Transfer is difficult because it depends on how knowledge is represented, contextualized, and abstracted (Barnett and Ceci, 2002). In the System 3 Method, transfer is the operational sign that learning has exceeded immediate performance and become usable for future thought.

A worked contrast can clarify the instructional mechanics. Consider a child reading a short scientific text about ecosystems. In a passive condition, the child reads the text and answers comprehension questions. In a standard generative condition, the child writes a summary or draws a food chain. In a System 3 condition, the teacher or system first activates intuitive orientation with a concrete image or puzzle, such as “Why can removing one animal change an entire ecosystem?” The learner then chooses one of three content-relevant ideas as most important, explains why it seemed important, draws or states a causal model, receives feedback on accuracy, revises the model, answers a retrieval question based on the chosen idea, and later applies the concept to a new ecosystem. The distinctive prediction is not merely higher enjoyment. It is that the System 3 condition should produce stronger delayed transfer and better metacognitive calibration because the learner's choice is content-relevant, articulated, verified, integrated, and retrieved.

This example also shows what would not count as System 3. A colorful story about an unrelated animal might increase interest but function as a seductive detail. A free-choice activity without verification might increase agency but not understanding. A quiz without reflection might measure recall without training metacontrol. The System 3 task changes what the teacher does: the teacher designs bounded relevance options, requires articulation, supplies feedback, prompts revision, and preserves process traces. It changes what the student produces: not only an answer, but a choice rationale, an explanation, a revision, a confidence judgment, and a transfer attempt.

## 6 Developmental and domain calibration: from intuitive play to creative metacontrol

The System 3 Learning Cycle must be calibrated to development, domain knowledge, and expertise. If deep learning depends on iterative integration of intuition, reason, feedback, and metacognitive regulation, pedagogy cannot impose the same degree of abstraction, autonomy, or explicit self-monitoring at every age or in every domain. A mature adult learning quantum mechanics, statistics, or a new language may have strong general metacognitive abilities but novice domain knowledge. In System 3 terms, development and expertise are separable constraints: age influences the maturity of executive and metacognitive capacities, while domain knowledge shapes what can be generated, verified, and integrated.

In early childhood, System 3 learning should begin with play, objects, movement, images, stories, and affectively meaningful situations. Young children show powerful capacities for exploration, causal learning, and hypothesis testing (Gopnik, 2012), but reflective control, verbal justification, and explicit metacognitive monitoring remain limited and uneven. System 3 experiences at this stage should therefore be concrete and tightly scaffolded: the child touches, chooses, predicts, draws, narrates, acts, or compares before being asked to justify abstractly. Guided play is especially compatible with this stage because it combines child autonomy with adult structure (Weisberg et al., 2016). Intuitive orientation and Generation are strong entry points; Reflection and Metacontrol require prompts, modeling, feedback, and environmental design.

During middle childhood, learners become increasingly able to compare ideas, inhibit first responses, hold goals in mind, and shift strategies. Executive functions such as working memory, inhibitory control, and cognitive flexibility develop substantially across childhood and adolescence (Best and Miller, 2010; Diamond, 2013). This allows System 3 activities to become more explicit: children can explain why an idea is plausible, how they could test it, what changed after feedback, and which strategy helped them. At the same time, metacognitive development is gradual (Kuhn, 2000; Roebers, 2017). Prompts should remain brief, concrete, and tied to observable action.

Adolescence and adulthood permit further expansion of System 3 cycles. Learners become increasingly capable of abstract reasoning, counterfactual thinking, identity-linked reflection, and self-conscious evaluation. Metacognitive ability shows a prolonged developmental trajectory through adolescence (Weil et al., 2013), and creative cognition develops unevenly across divergent thinking and insight (Kleibeuker et al., 2013). For older learners, System 3 pedagogy can include hypothesis construction, argumentative writing, ethical dilemmas, design challenges, and scientific inquiry. Yet adult novices still need domain scaffolding: they may regulate effort well but lack the conceptual criteria needed to verify or integrate ideas.

This developmental logic can be summarized in one principle: the less mature or less domain-prepared the learner, the more concrete, externally scaffolded, and criteria-rich the System 3 cycle should be; the more mature and domain-prepared the learner, the more abstract, self-regulated, and transferable the cycle can become. Developmental calibration protects the method from premature openness and premature rationalism. It strengthens generation without sacrificing reflection, protects imagination without abandoning verification, and gradually transforms guided participation into creative metacognitive agency.

## 7 Assessment: GRIM traces and NUD learning profiles

Assessment in the System 3 Method must be consistent with its theory of learning while avoiding circularity. Deep learning should not be inferred simply because a learner engaged in a System 3-like process. It should be anchored to independently observable outcomes: delayed recall, explanation accuracy, transfer to structurally related cases, confidence calibration, quality of revision after feedback, and the ability to select an appropriate strategy in a new context. Process indicators explain how the outcome may have emerged; they do not replace outcome evidence.

The method proposes a three-layer model of assessment. The first layer is academic mastery. Students must demonstrate accurate reading, conceptual understanding, mathematical reasoning, scientific explanation, argument quality, or disciplinary skill. A System 3 pedagogy should not confuse originality with understanding. A learner's explanation may be personally meaningful and novel yet inaccurate, while another learner may reproduce a correct answer without integrating it meaningfully. Academic mastery remains necessary but insufficient.

The second layer is GRIM process tracing. Generation can be observed through the number, variety, and relevance of questions, hypotheses, examples, representations, or solution paths the learner produces. Reflection can be observed through justification, comparison, error detection, evidence use, and revision. Integration/Iteration can be observed when the learner connects images, narratives, concepts, rules, feedback, and prior knowledge into a more coherent representation. Metacontrol can be observed when the learner monitors confidence, confusion, effort, strategy, progress, and the need to revise. Time can be observed through improvement across repeated cycles rather than isolated performance. These traces are not intended to label students as globally “creative” or “uncreative”; they identify where support is needed.

The third layer is the NUD learning profile: novelty, usefulness, and diversity. In educational contexts, novelty should be judged primarily as mini-c or learner-relative novelty: a personally new connection, representation, question, or solution relative to the learner's prior state (Kaufman and Beghetto, 2009). Usefulness refers to whether the contribution advances the current learning objective, improves explanation, solves a problem, or opens a productive line for future inquiry. Some ideas may not be immediately useful as final answers but may be useful as exploratory steps. Diversity refers to whether the learner explores multiple categories, strategies, representations, examples, or perspectives rather than repeatedly varying one familiar path.

A simple NUD rubric could use four anchors. Novelty: 0 = copied or routine; 1 = minor variation; 2 = personally new connection; 3 = personally new and conceptually generative connection. Usefulness: 0 = irrelevant or inaccurate; 1 = partially related; 2 = supports the target understanding; 3 = supports understanding and enables transfer or further inquiry. Diversity: 0 = single path; 1 = superficial variations; 2 = multiple representations or strategies; 3 = multiple representations coordinated with evidence and constraints. Reliability would require trained raters, domain-specific exemplars, and inter-rater agreement, rather than unanchored impressions. Consensual assessment traditions in creativity research show why domain-relevant judgment matters (Amabile, 1982), while formative assessment research shows that criteria are most useful when they guide improvement rather than merely score products (Black and Wiliam, 1998; Panadero and Jönsson, 2013; Shute, 2008).

System 3 assessment also benefits teachers. Process traces help teachers see whether a student lacks generative options, verification criteria, integration, or metacognitive calibration. Thus, assessment functions as both a learner scaffold and a teacher-observation tool. When assessment captures only the final answer, the learner may learn to perform. When assessment captures the System 3 process, both learner and teacher can see how intuitive orientations, rational explanations, revisions, and transfers evolve over time.

## 8 Technology and AI as metacognitive scaffolds under the human gate

Technology is not the foundation of the System 3 Method, but it may become one of its most powerful implementation environments. The method is grounded in a theory of learning: deep understanding emerges through iterative integration of intuition, reason, feedback, and metacognitive control. Artificial intelligence can support this process only when it strengthens human capacities rather than replacing them. AI is pedagogically legitimate in the System 3 Method not as an autonomous teacher, but as a scaffold operating under a human gate.

The educational promise of AI is partly continuous with the aspiration to approximate tutoring responsiveness at scale (Bloom, 1984). AI systems can contribute adaptive feedback, differentiated explanations, question generation, practice design, and process tracing. Such systems may also support generalizability and scalability when designed around stable, interpretable measurements and implementation contexts, an issue emphasized in modelability work in learning analytics (Xu et al., 2026b). However, reviews of AI in education show that pedagogical integration remains uneven (Zawacki-Richter et al., 2019), and large language models require critical literacy, fact checking, human oversight, and pedagogical strategy (Kasneci et al., 2023).

Within the System 3 Method, AI can serve four functions. It can act as a generative scaffold, expanding the learner's possibility space through examples, analogies, counterexamples, stories, prompts, or alternative representations. It can act as an adaptive critic, helping learners compare explanations against evidence, constraints, and disciplinary criteria. It can act as a metacognitive mirror, prompting learners to ask why they chose a path, what evidence supports it, and how their confidence changed. Finally, it can act as process memory: a record of choices, explanations, revisions, feedback, confidence judgments, and transfer attempts across time. These functions extend computer-based scaffolds for self-regulated learning and metacognition (Azevedo and Hadwin, 2005).

However, the same capabilities introduce risks. Generative AI can increase individual fluency while reducing collective diversity of outputs (Doshi and Hauser, 2024). It may also produce fluent but false explanations, reinforce bias, create dependency, or encourage students to outsource effort. Recent preprint evidence suggests that AI assistance can reduce persistence and impair later independent performance, even when it improves immediate assisted performance (Liu et al., 2026). These risks directly threaten System 3 learning. A learner who receives answers without generating, verifies without understanding, or reflects only through machine-produced language is not developing System 3 capacity.

For this reason, the System 3 Method requires a human gate: learners, teachers, and the pedagogical community retain responsibility for aims, interpretation, verification, values, and final judgment. In previous work on Generative System 3, creative AI was described as requiring a generator, a critic, and an adaptive gain controller (Chávez-Autor, 2025). Translated into education, these roles should progressively become internalized by learners. Teachers can model the roles explicitly: “generate several possibilities,” “criticize them against evidence,” and “adjust the search when the path is too narrow or too diffuse.” AI may support that process, but it should not occupy the roles permanently. The goal is not faster answer provision; it is the development of learners who can generate, evaluate, regulate, and transfer independently.

## 9 Relation to Montessori, Waldorf, and learner-centered traditions

The System 3 Method should not be presented as a rejection of classical learner-centered pedagogies. Rather, it can be understood as a next-generation cognitive-metacognitive framework that shares several of their deepest concerns while translating them into a more explicit architecture of intuition-reason integration, creative metacognition, process assessment, and responsible technology mediation. Its ambition is not to replace historical traditions by slogan, but to provide a testable grammar for what many powerful educational traditions have intuited: children learn deeply when they act, imagine, choose, construct, and progressively regulate their own understanding.

Montessori education is an especially relevant comparison. The Montessori tradition emphasizes the prepared environment, hands-on materials, self-directed activity, long periods of concentration, and the teacher's role as observer and guide (Montessori, 1912; Marshall, 2017). Empirical and review work has identified Montessori education as a complex package involving structured materials, self-directed engagement, active exploration, and developmental aims that extend beyond conventional academic attainment (Lillard and Else-Quest, 2006; Marshall, 2017). The System 3 Method shares with Montessori the view that autonomy and environment matter. However, it differs in what it makes explicit. Montessori prepares the environment; the System 3 Method specifies the cognitive-metacognitive cycle that the environment should support: intuitive orientation, generative engagement, rational articulation, verification, integration, metacognitive reflection, and transfer.

Waldorf education offers a second important comparison. Waldorf traditions emphasize imagination, narrative, artistic activity, rhythm, developmental sensitivity, and the education of the whole child (Steiner, 1996; Dahlin, 2017; Ullrich, 1994). These concerns resonate strongly with the System 3 Method, particularly its claim that affective, embodied, narrative, and intuitive orientations are entry points into meaning. However, the System 3 Method reframes imagination as part of a regulated creative learning cycle. Narrative, symbol, art, and image are educationally powerful not simply because they are expressive, but because they can activate intuitive salience, generate possibilities, support conceptual integration, and become objects of reflective verification.

The method also relates to constructivist, inquiry-based, and active-learning traditions. Like them, it rejects the idea that learning is passive reception. However, it is not a theory of minimal guidance. Research on discovery learning suggests that unassisted discovery often underperforms more guided forms, whereas enhanced discovery with feedback, scaffolding, worked examples, and elicited explanations can support stronger outcomes (Alfieri et al., 2011; Kirschner et al., 2006; Lazonder and Harmsen, 2016). Defenses of problem-based and inquiry learning similarly emphasize that these approaches are effective when scaffolded rather than when learners are left unsupported (Hmelo-Silver et al., 2007). The System 3 Method is therefore best understood as a pedagogy of structured freedom: learners generate and explore, but always within cycles of articulation, feedback, integration, and metacognitive control.

This positioning clarifies the distinctive contribution of the System 3 Method. Montessori foregrounds prepared environments and self-directed work. Waldorf foregrounds imagination, development, and holistic formation. Constructivist and inquiry traditions foreground active meaning-making. The System 3 Method attempts to integrate these strengths into a single psychological architecture: learning as repeated, developmentally and domain-calibrated integration of intuition and reason under implicit and explicit metacognitive control.

## 10 Boundary conditions and testable propositions

Every ambitious pedagogical framework requires boundary conditions. The System 3 Method is not a claim that all autonomy improves learning, all creativity is beneficial, all reflection improves regulation, or all technology-supported personalization leads to understanding. Its central claim is narrower: deep learning is strengthened when learners repeatedly integrate intuitive salience, rational articulation, feedback, and metacognitive regulation into transferable understanding. This claim can fail under identifiable conditions.

The first boundary condition is instructional load. A System 3 learning environment should open a space of possibility but not leave novices without criteria. The issue is not that open environments always impose high cognitive load; learners may attend narrowly even in open contexts. The risk is that unbounded exploration can create extraneous search demands when learners lack schemas or standards for evaluating alternatives. Cognitive load theory warns that poorly structured problem solving can consume resources needed for schema acquisition (Sweller, 1988), and critiques of minimally guided instruction show that novices often require explicit guidance, worked examples, feedback, and scaffolding (Kirschner et al., 2006). System 3 pedagogy is therefore structured freedom.

The second boundary condition is choice quality. Choice can support motivation and performance, but too many options or poorly structured options can become demotivating or burdensome (Iyengar and Lepper, 2000; Patall et al., 2008). In System 3 terms, a choice is educationally valuable only when it becomes part of a learning cycle. A learner must not merely select; the learner must elaborate, test, integrate, and reflect. The third boundary condition is metacognitive validity. Learners often misjudge their own learning, and short-term performance can be mistaken for durable understanding (Bjork et al., 2013; Soderstrom and Bjork, 2015). System 3 reflection must therefore be concrete, evidence-linked, and calibrated to developmental and domain expertise.

The research agenda must test components, not merely the whole framework. A first line of studies should compare matched instructional conditions: passive exposure; generative activity without verification; verification without learner generation; metacognitive prompting without revision; and full System 3 cycles. Outcomes should include delayed recall, near and far transfer, explanation accuracy, confidence calibration, and persistence. A second line should use mediation analyses to test whether transfer advantages are explained by integration quality, not only by interest or time on task. A third line should test moderation by age, domain knowledge, prior achievement, and metacognitive skill. A fourth line should compare AI answer-provision tools with AI scaffolds that require students to generate, verify, and revise.

Model revision should be explicit. If generative activity improves recall but not transfer, the integration component may be insufficient. If reflection prompts increase effort without improving calibration, metacognitive scaffolds should be simplified or moved earlier/later in the cycle. If AI scaffolds increase fluency while reducing persistence or diversity, the human gate has failed. If System 3 cycles do not outperform well-matched active-learning conditions on delayed transfer after controlling for time, feedback, and domain knowledge, the framework must be narrowed to contexts requiring conceptual change, creative transfer, or self-regulated inquiry. Future work should therefore combine laboratory experiments, classroom field trials, and design experiments capable of refining complex educational interventions in real settings (Brown, 1992; Koedinger et al., 2013). The System 3 Method is not only a pedagogical proposal; it is a research program whose strongest claims should be tested, constrained, and improved through evidence.

## 11 Discussion: toward a pedagogy of creative metacognition

This Conceptual Analysis proposed the System 3 Method as a pedagogical framework for deep and transcendent learning, understood as learning that exceeds immediate task performance by becoming meaningful, transferable, self-regulated, and formative for future thought. Its central claim is that education should train not only what learners know, but also how learners integrate intuition, reason, feedback, and metacognitive control across time. The method does not reject meaningful learning, generative learning, active-constructive engagement, metacognition, curiosity-driven learning, or knowledge integration. It attempts to synthesize them into a process-level architecture organized around System 3 integration.

The distinctive contribution of the System 3 Method is to place creative metacognitive integration at the center of educational design. Learning is not treated as passive exposure, isolated activity, unbounded exploration, or mere preference expression. It is treated as a recurrent cycle in which learners notice intuitive relevance, generate or reconstruct possible meanings, articulate them rationally, verify them through feedback and evidence, integrate revisions, reflect on their own process, and transfer understanding to new contexts. This cycle gives practical form to the guiding proposition of the article: learners remember more deeply what they help make meaningful. The claim is not that subjective meaning replaces academic standards, but that academic understanding becomes more durable when learners participate in its content-relevant construction, evaluation, and integration.

GRIM^T^ and NUD provide the method's design and assessment language. GRIM^T^ describes the process structure through which System 3 learning unfolds. NUD extends assessment beyond final correctness by capturing the creative quality of learning trajectories (Chávez-Autor, 2025). Together, these constructs allow educators and researchers to ask whether a learning environment expands possibilities, requires verification, integrates intuitive and rational codes, strengthens metacognitive regulation, and preserves diversity across repeated cycles.

Several limitations follow. First, the article is conceptual and requires empirical validation. Second, the constructs proposed here require operational refinement across domains, ages, and cultures. Third, process traces should support metacognition, not convert learning into surveillance. Fourth, the balance among Generation, Reflection, Integration, and Metacontrol must be developmentally and domain calibrated. A poorly designed System 3 environment could overload novices, reward superficial originality, or add reflection prompts without improving regulation.

The promise of the System 3 Method is that education can teach learners not only to know more, but to participate more intelligently in the construction, evaluation, integration, and transformation of what they know.
